# LRP-1-mediated intracellular antibody delivery to the Central Nervous System

**DOI:** 10.1038/srep11990

**Published:** 2015-07-20

**Authors:** Xiaohe Tian, Sophie Nyberg, Paul S. Sharp, Jeppe Madsen, Nooshin Daneshpour, Steven P. Armes, Jason Berwick, Mimoun Azzouz, Pamela Shaw, N. Joan Abbott, Giuseppe Battaglia

**Affiliations:** 1 Department of Chemistry, University College London, London, UK; 2 The MRC/UCL Centre for Medical Molecular Virology, University College London, London, UK; 3 Department of Biomedical Science; 4 Department of Chemistry, University of Sheffield, UK; 5 Sheffield Institute for Translational Neuroscience (SITraN), University of Sheffield, UK; 6 Department of Neuroscience, University of Sheffield, UK; 7 Department of Psychology, University of Sheffield, UK; 8 Institute of Pharmaceutical Science, King’s College London, UK

## Abstract

The blood-brain barrier (BBB) is by far the most important target in developing new approaches to improve delivery of drugs and diagnostic tools into the Central Nervous System (CNS). Here we report the engineering of pH- sensitive polymersomes (synthetic vesicles formed by amphiphilic copolymers) that exploit endogenous transport mechanisms to traverse the BBB, enabling delivery of large macromolecules into both the CNS parenchyma and CNS cells. We achieve this by targeting the Low Density Lipoprotein Receptor-Related Protein 1 (LRP-1) receptor. We show that LRP-1 is associated with endothelial transcytosis that does not involve acidification of cargo in membrane-trafficking organelles. By contrast, this receptor is also associated with traditional endocytosis in CNS cells, thus aiding the delivery of relevant cargo within their cytosol. We prove this using IgG as a model cargo, thus demonstrating that the combination of appropriate targeting combined with pH-sensitive polymersomes enables the efficient delivery of macromolecules into CNS cells.

The fundamental role of the Central Nervous System (CNS, which comprises the brain and spinal cord) in controlling body functions is associated with its isolation from the rest of the body. A tight network of membrane barriers controls the transport of nutrients, metabolites and signalling molecules in and out of the CNS, with permeability and trafficking uniquely tailored to the CNS. These barriers include the blood-brain barrier (BBB) at the brain microvascular endothelium, and the blood-cerebrospinal fluid barrier (BCSFB) at the choroid plexus and the arachnoid epithelium^1^. Of these, the BBB is arguably the most important barrier as it allows access to almost all components of the CNS, being the largest in surface area and the one with the shortest diffusion distance to individual cells of the CNS parenchyma^1^.

The BBB is not only an anatomical barrier, but also acts as a metabolic barrier to very precisely control transport between the blood and the CNS. The BBB consists of specialised and highly polarised vascular endothelial cells, which in contrast to peripheral endothelia lack fenestrations, show low expression of immune cell adhesion molecules, and express extremely tight ‘tight junctions’ that lead to severe restriction of paracellular transport. Brain endothelial cells also control transcellular transport by the expression of specialised molecular transporters at the apical and basolateral membranes, and by limiting vesicular transport via transcytosis to relatively few ligands^2^. These unique phenotypic functions are the result of the interaction with CNS-resident pericytes^3^, astrocytes^2^, microglia and neurons^1^. Together with the endothelial cells, these cell types form the so-called ‘neurovascular unit’. This highly regulated and relatively impermeable barrier is a major obstacle for developing new therapeutic approaches to treat neurological diseases^4^,^5^,^6^, and engineering new probes to study the complexity of the CNS.

One approach to address this problem is to develop a carrier that exploits endogenous transcytosis routes to traverse the BBB, enabling the delivery of therapeutics into the CNS without disrupting homeostasis. Transcytosis involves the formation of membrane-bound vesicles on the apical side of endothelia that are quickly moved to the basolateral side where the vesicles fuse with the membrane, releasing the cargo within the CNS^7^. Such a transport mechanism enables the movement of macromolecules, including several proteins and lipoproteins. Furthermore, it is often used by pathogens to gain entry to the CNS^8^. Achieving transcytosis by targeting endogenous transport systems of the BBB is a highly selective and non-invasive delivery mechanism for the CNS, which should be particularly relevant for macromolecular payloads. Several receptors for receptor-mediated transcytosis (RMT) are highly expressed on the endothelial cells that form the BBB, including the low-density lipoprotein receptor-related protein 1 (LRP-1), insulin receptor (IR), transferrin receptor (TfR) and others^9^,^10^,^11^,^12^. Previous efforts using ligand-functionalised carriers, including solid lipid nanoparticles^13^, liposomes^14^, dendrimers^15^ and micelles^16^, have been reported to facilitate delivery across the BBB. However, even in the best cases the delivery efficacy has not led to clinical translation, hence more effective strategies to improve CNS delivery are still required. Furthermore, traversing the CNS is not the only challenge associated with designing effective therapeutics. Often the cargo requires delivery into specific CNS sub-compartments, or even entry into CNS resident cells to access their machinery more effectively. Here we use *in vitro*, *in vivo* and *ex vivo* approaches to examine the combination of transcytosis-targeting motifs with pH-sensitive polymersomes that have been previously demonstrated to facilitate cellular delivery^17^,^18^,^19^,^20^. We use an established 3D transwell co-culture setup to mimic the BBB *in vitro*. This simplistic transwell setup has been widely reported to mimic the BBB phenotype^21^,^22^ and is suitable for studying essential brain endothelial cell functions such as permeability and transcytosis. This model has allowed us a simplified view of interaction of polymersomes at the BBB, focusing primarily on endothelial cell functions.

Polymersomes are synthetic vesicles formed by the self-assembly of amphiphilic copolymers in water^23^. Over the last ~eight years, we have studied the self-assembly of biocompatible diblock copolymers which contain a hydrophobic pH-sensitive poly(2-(diisopropylamino)ethyl methacrylate) (PDPA) block that has a pK_a_ of ≈ 6.4. Once PDPA-based polymersomes enter cells via receptor-mediated endocytosis, they are trafficked to sorting endosomes where the reduction in local pH triggers polymersome dissociation. If the local pH is below the pK_a_, the tertiary amine groups on the PDPA chains become extensively protonated, rendering the PDPA chains hydrophilic in their weak cationic polyelectrolyte form. As a consequence, polymersomes disintegrate to produce many individual copolymer chains, with a resulting dramatic increase in the number of species. This, in turn, triggers a rise in osmotic pressure that temporarily lyses the endosomal membrane, permitting the release of encapsulated substances into the cell cytosol. This approach enables the cytosolic delivery of several payloads, including anticancer drugs^24^,^25^ and antibiotics^26^ as well as nucleic acids^18^,^27^, proteins^27^,^28^ and many other molecular species^29^,^30^. PDPA-based polymersomes can be constructed using several hydrophilic blocks and in principle functional peptides can be introduced to control cell specificity. Herein we use PDPA-based polymersomes for which the permanently hydrophilic stabiliser block is either poly[2-(methacryloyloxy)ethyl phosphorylcholine] (PMPC) or poly[oligo(ethylene glycol) methyl methacrylate] (POEGMA). These were further functionalised with relevant peptides through the use of a protected maleimide-functional ATRP initiator^31^. The resulting polymersomes contain two peptide sequences already proven to facilitate BBB transport: LRP-1 targeting Angiopep-2^15^,^16^,^32^ and Rabies Virus Glycoprotein (RVG)^14^,^33^, while the PMPC block has the ability to target Scavenger Receptors class B1 (SR-B1) receptors^24^. Angiopep-2 has already been extensively utilised to target LRP-1 receptors, while it is still unknown whether RVG transcytosis can be facilitated. We have recently shown that PMPC targets both SR-B1 and CD36, with the former being targeted for BBB crossing^34^. Using ligand-functionalised polymersomes is particularly advantageous as their properties can be controlled at both the molecular and supra-molecular level, allowing fine-tuning of the polymersome size, surface chemistry, and topology^20^,^35^. The overall aim of our study was to develop ligand-functionalised polymersomes to facilitate CNS delivery, and more importantly to demonstrate efficient intracellular delivery of a model antibody into CNS resident cells.

## Results

### Polymersome 2D and 3D *in vitro* screening

**Figure 1:**
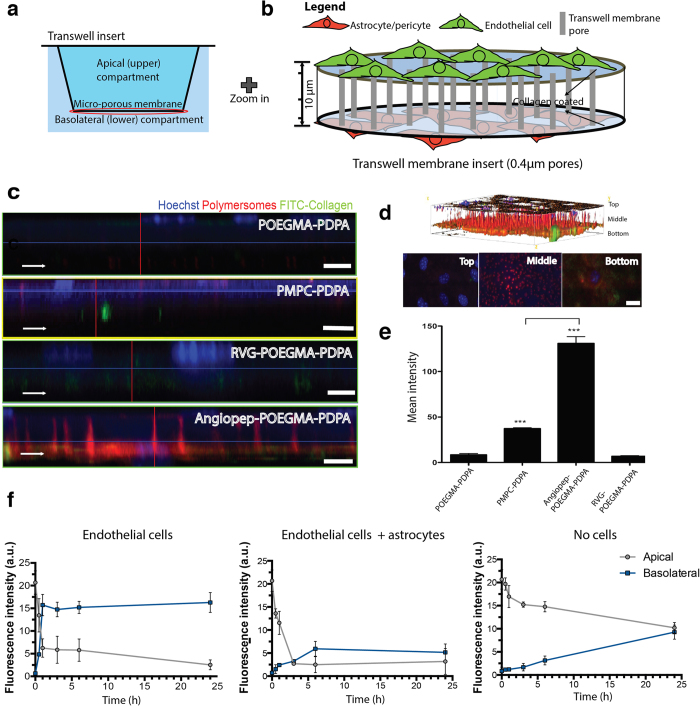
Screening functionalised polymersomes on the *in vitro* BBB models. (**a**) Schematic representation of the transwell system, consisting of a transwell insert with a base made of microporous membrane separating the well into upper and lower compartments. (**b**) Schematic representation of cells grown on both sides of transwell insert membrane coated with rat-tail collagen. (**c**) 3D Z-stack confocal micrograph of transwell insert membrane plus cells treated with polymersomes. (**d**) Micrograph sections of top/middle/bottom view of insert membrane and cells treated with A-EP polymersomes and (arrow) 3D volume viewer. (**e**) Fluorescence analysis of transwell insert microporous membrane and cells after different polymersome treatments. (**f**) Transwell permeability assay, timecourse of fluorescence from A-EP polymersomes in cell media of upper and lower compartments. Scale bars 20 μm. One-way ANOVA was used for statistical analysis for n = 3 independent experiments, p < 0.005. Error bars: SEM.

Polymersomes including POEGMA-PDPA (EP), PMPC-PDPA (Supplementary Fig. 1a and 1b) and peptide-functionalised EP were prepared via a pH-switch method; this is a ‘bottom-up’ self-assembly process that can be precisely manipulated, as reported elsewhere^35^,^36^. The resulting polymersomes had a mean diameter of 100 nm (Supplementary Fig. 1c) and transmission electron microscopy (TEM) studies confirmed their vesicular morphology (Supplementary Fig. 1d). Further physicochemical characteristics, and their uptake by the mouse brain endothelial cell line bEnd.3, can be found in Supplementary Fig. 1 and Supplementary Fig. 2. The most effective formulations for cellular uptake were further tested for transcytosis efficiency. To do so, we employed a 3D *in vitro* BBB model where brain endothelial cells were cultured on collagen-coated trans-well microporous filter inserts in which the upper compartment is connected to the lower (basolateral) compartment via 0.4 μm pores through the filter (Fig. 1a). The underside of the filter facing the lower compartment was used to culture astrocytes (mouse astrocyte cell line) and/or ‘pericytes’ (mouse mesenchymal stem cell line with pericyte-like properties) to induce a more effective BBB^37^. Using this 3D model, we were able to distinguish formulations that can enter brain endothelial cells via endocytosis from those that target receptors associated with transcytosis, since the latter process involves active transport across the cell layer and consequent accumulation in the microporous membrane and/or basolateral compartment (Fig. 1b).

Several different formulations of fluorescently labelled polymersomes were added to the upper compartment of the 3D BBB model consisting of endothelial/astrocyte co-culture (refer Supplementary Fig. 3 for transendothelial electrical resistance, TEER), and the polymersome location was assessed after 2 hours. The Z-stacks acquired by confocal microscopy showed whether polymersomes were retained by the endothelial cells (top side) or were shuttled across to the pores or the astrocytes on the lower filter surface (Fig. 1c and Supplementary Fig. 4). As expected, pristine EP showed “stealth” properties with very little cellular uptake being observed (Fig. 1c). By contrast, PMPC-PDPA polymersomes, which are taken up avidly by many types of cells^20^, were internalised by the endothelial cell layer and concentrated within the cytosol, rather than the transwell filter pores (Fig. 1c). We now know that this is the result of the high affinity of PMPC towards SR-B1 and CD36^24^. Although the SR-B1 receptor has been associated with transcytosis, most of the polymersomes were actually retained within the endothelial cells. Finally, despite positive uptake by endothelial cells being identified in a 2D assay, RVG-POEGMA-PDPA polymersomes were not internalised efficiently in the 3D culture environment (Fig. 1c) and, more importantly, were not transported across the endothelial cell layer. Interestingly, the Angiopep-2-EP (A-EP) treated group initially showed less uptake by bEnd.3 cells compared to PMPC-PDPA polymersomes, with a strong fluorescence signal observed within the transwell filter pores (Fig. 1c). This suggests that most of the polymersomes were transported across the endothelial monolayer during the incubation time. This evidence for transcytosis was reinforced by the presence of polymersomes in the FITC-conjugated collagen coating of the filter and within the astrocytes growing on the lower surface of the filter. Transwell filter membrane fluorescence (quantified over three independent experiments) revealed that A-EP polymersomes were shuttled across the bEnd.3 cells more effectively than all other formulations (Fig. 1e). Images reconstructed into 3D renders illustrated this result in more detail (Fig. 1d and Supplementary Fig. 5).

**Figure 2:**
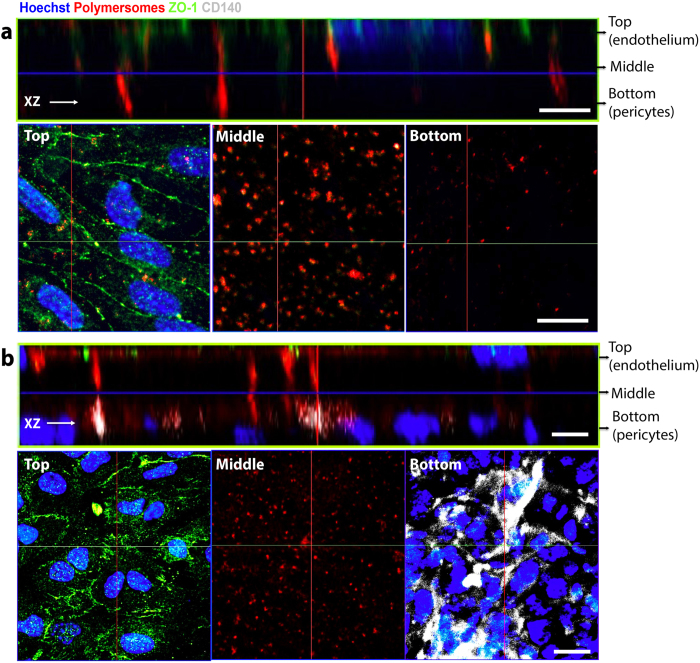
*In vitro* BBB model assessment with A-EP polymersomes. (**a**) bEnd.3 monolayer on transwell insert treated with A-EP polymersomes (3D z-stack and top/middle/bottom section micrographs). (**b**) bEnd.3 co-cultured with pericytes on transwell insert treated with A-EP polymersomes (3D z-stack and top/middle/bottom section micrographs). Scale bars 20 μm.

Quantification of A-EP fluorescence in the culture media of both compartments revealed rapid apical-to-basolateral compartment transport, which was markedly faster in the presence of cells relative to the cell-free control (Fig. 1f). Most of the polymersome signal in the upper compartment had disappeared by 60 minutes, suggesting that polymersome transport occurs via an active process. The signal from the lower compartment slowly reached a plateau in the astrocyte co-culture; this may indicate intracellular uptake by the astrocytes, rather than the polymersomes moving freely into the lower compartment medium as observed the endothelial cell monoculture. With counter-staining of the 3D model for endothelial tight junction protein Zonula Occludens 1 (ZO-1) as an indicator of barrier formation and integrity, A-EP was found to penetrate the endothelial monolayer across its whole surface (Fig. 2a). In fact, transcytosis was still observed after enhancing the tightness of our 3D model by the introduction of pericytes^38^, as confirmed by the increased TEER and positive expression of PDGFR-β/CD140 marker (Fig. 2b and Supplementary Fig. 6) hence unlikely to be via paracellular transport through the tight junctions. Finally, a ‘reverse’ model with endothelial cells on the underside of the transwell filter (pericytes on top, α-SMA, smooth muscle actin) also showed effective transcytosis (Supplementary Fig. 7). Taken together, these data indicate the transcellular movement of polymersomes across a tight endothelial layer via an active transport mechanism, rather than passive diffusion.

### Exploring A-EP transcytosis mechanisms *in vitro*

**Figure 3:**
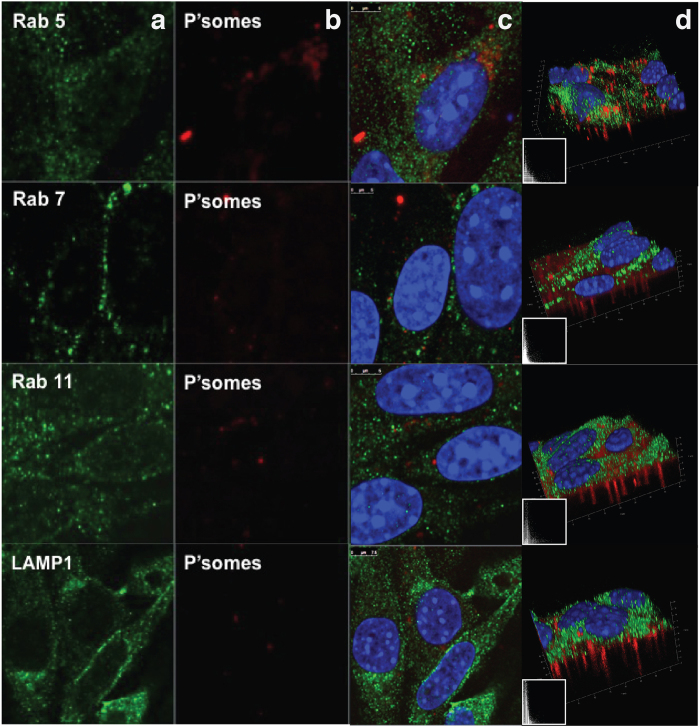
Co-localisation study of A-EP polymersomes with Rab5, Rab7, Rab11 and LAMP1 via immunocytochemistry in transwell inserts. (**a**) Expression of Rabs. (**b**) Polymersome location in a cross-section. (**c**) Overlaid antibody and polymersome stacks with DAPI. (**d**) Z-stacks reconstructed into 3D images from a and b and their co-localisation profiles (insert).

**Figure 4:**
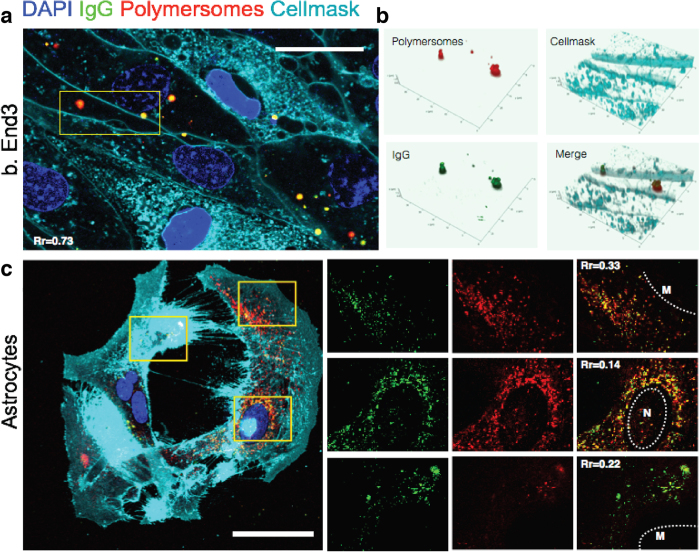
Study of A-EP transcytosis mechanisms *in vitro.* (**a**) Co-localisation study of A-EP polymersomes with encapsulated IgG in bEnd.3 cells. (**b**) Z-stacks reconstructed into 3D images from selected region in a. (**c**) In astrocytes, IgG encapsulated within A-EP polymersomes show a different endocytic interaction profile compared to bEnd.3 cells, with evidence for release into the cytosol. The small panels show higher magnification views of the areas selected, with IgG (green) and A-EP (red) showing a low colocalisation profile. Rr: Pearson’s correlation coefficient. M: membrane, N: nucleus. Scale bars 10 μm.

Next, we examined the intracellular mechanism of LRP-1 mediated transcytosis. Consistent with previous reports, we observed a high degree of association of the Angiopep-2-functionalised polymersomes with LRP-1 receptors (Supplementary Fig. 8). Bearing in mind the kinetic data, LRP-1 most likely mediates the transcytosis of polymersomes in bEnd.3 cells. Immunocytochemistry was then used to assess co-localisation of polymersomes with Rab5, Rab7, Rab11 and LAMP1. These markers correspond to early endosomes, late endosomes, recycling endosomes and lysosomes, respectively. Surprisingly, polymersomes were not associated with any of these membrane-trafficking markers after 15 minutes (Fig. 3), with a very low Pearson’s coefficient indicating almost no co-localisation (Fig. 3d). Z-stacks reconstructed into 3D images further illustrate the extensive presence of the polymersomes within the transwell membrane pores, showing that polymersomes have undergone transcytosis. These results are unexpected, and imply a non-acidifying pathway for endothelial transcytosis. To test this hypothesis, we encapsulated IgG into Angiopep-2-functionalised polymersomes and investigated their behaviour *in vitro*. During the transcytosis events, the IgG signal is almost perfectly co-localised with that of the polymersomes (Fig. 4a,b). This indicates that the polymersomes remain intact during transcytosis, i.e. dissociation does not occur since the polymersomes do not enter acidified compartments. Thus the counter-hypothesis that transcytosis occurs via a series of membrane-bound acidifying organelles such as endosomes is not observed for this receptor, as the pH-sensitive polymersomes would quickly release their IgG cargo under such conditions^28^. By contrast, IgG-loaded polymersomes exhibit a different endocytic interaction within astrocytes (Fig. 4c). In this case the cargo and polymersome signals are not co-localised, suggesting intracellular delivery of the IgG to the cytosol as reported previously for similar polymersomes and several cell types^28^. These data strongly suggest that, although LRP-1 is associated with an endocytic event in both endothelial and glial cells, intracellular sorting differs significantly between the two cell types. In polarised endothelial cells, LRP-1 controls transcytosis and transport, while in the glial cells the same receptor is associated with canonical endocytic degradation. This enables the same ligand (Angiopep-2) to be used to both facilitate transport across the BBB and to achieve intracellular delivery within CNS resident cells. Taken together, these data suggest that LRP-1 mediates polymersome transcytosis across the endothelial cell *in vitro* by avoiding acidic degradation, with no association with endosomes or lysosomes being observed. These findings are significant not only for delivering cargo *in vitro* or *in vivo*, but also because they highlight a gap in our understanding of how receptor-mediated transcytosis occurs in polarised endothelial cells. Nevertheless, we have demonstrated the successful transcytosis of IgG-loaded polymersomes in a 3D model of the BBB.

### Tissue distribution of intravenously administered polymersomes

**Figure 5:**
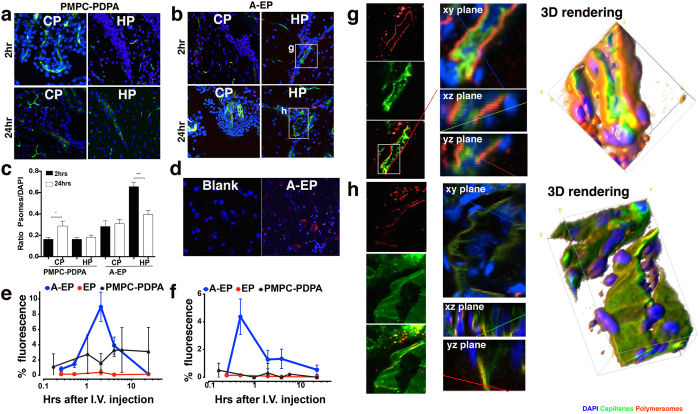
*Ex vivo* assessment of polymersomes following *i.v.* injection in mice. (**a,b**) Confocal micrographs of choroid plexus (CP) and hippocampus (HP) extracted from mice after 2 hr and 24 hr after i.v. injection of PMPC-PDPA polymersomes (**a**), and A-EP polymersomes (**b**). (**c**) Fluorescence intensity analysis of uptake of PMPC-PDPA and A-EP polymersomes in CP and HP at 2 and 24 hours. (Y_value_ = Intensity_Psomes_/Intensity_DAPI_) (**d**). Immunofluorescence histology of spinal cord imaged 10 mins after i.v. injection of A-EP polymersomes (**d**). The blank is from untreated mouse. (**e,f**) Pharmacokinetics of A-EP, EP and PMPC-PDPA polymersomes in the brain (**e**) and the spinal cord (**f**). The data were obtained by normalising the Total Radiant Efficiency (TRE) [p/s] / [μW/cm^2^] measured in each organ by the TRE measured in all organs. The TRE was measured using an IVIS spectrum by imaging whole organs excised at the different time points and post saline perfusion Error bars: SEM, n = 5. (**g,h**) Details of *in vivo* BBB crossing of A-EP polymersomes. The micrographs were selected from the sections in (**b**) and show the detail of brain capillaries imaged by confocal laser scanning microscopy, and displayed as 2D sections and 3D rendering of HP sections 2 hr (**g**) and 24 hr (**h**) after i.v. injection of A-EP polymersomes.

To investigate the potential of Angiopep-2-functionalised polymersomes to penetrate the BBB *in vivo* and enter the brain parenchyma, A-EP, PMPC-PDPA and non-targeted EP polymersomes were administered intravenously (i.v.) in C57BL/6 mice. The brain distribution of the three formulations was then analysed in thin brain tissue sections, 2 h and 24 h after i.v. administration. Furthermore, to ascertain whether polymersomes entered brain parenchyma or remained associated with the capillary endothelia, the brain microvasculature was also labelled *in vivo* using fluorescein-conjugated lectins^39^. Detailed analysis of all brain slices showed that accumulation of PMPC-PDPA polymersomes was associated with the ventricular system, particularly the choroid plexus of the 4^th^ ventricle (CP; Fig. 5a) and in the hippocampal region (HP; Fig. 5a). Polymersome accumulation increased from 2 h to 24 h both in CP (Fig. 5a) and HP (Fig. 5a), and showed a close association with capillary endothelium suggesting that SR-B1 targeting only results in polymersome entry into endothelia but not effective transcytosis into parenchyma. This could be due to injected polymersomes quickly circulating to choroid plexus vasculature, crossing the fenestrated endothelium (via open tight junctions, fenestrations or non-specific fluid-phase transcytosis), and finally accumulating in CP stroma or endocytosis by CP epithelial cells. The presence of polymersomes in the choroid plexus is compatible with entry across the leaky fenestrated endothelium of the CP, and accumulation within the extracellular fluid of the CP or within epithelial cells that constitute the blood-cerebrospinal fluid barrier^40^,^41^. Further studies are underway to investigate this.

Compared to PMPC-PDPA, A-EP fluorescence was strong two hours after administration in most areas of the brain, and we chose CP and HP (Fig. 5b) for further comparison and analysis. Unlike PMPC-PDPA which remained closely associated with the capillaries, most of the A-EP material appeared distributed within deeper brain tissues. Quantification of the confocal micrographs focusing on the CP and HP regions at different time-points (Fig. 5c) indicated that PMPC-PDPA polymersomes showed less entry into brain parenchyma compared to A-EP polymersomes. Finally, it is worth noting that out of all three formulations, only A-EP showed detectable levels of fluorescence in spinal cord sections (Fig. 5d).

The biodistribution of polymersomes was further analysed by *ex vivo* quantitative fluorescence imaging, expressed as Total Radiant Efficiency (TRE) [p/s] / [μW/cm^2^] of the excised and perfused organs and blood samples. The overall organ distribution is shown in the supporting information figure S9 for the three polymersome formulations, using either the absolute TRE measured or their normalised data per total TRE measured across all the organs. The levels of pristine EP polymersomes observed within the liver, spleen and bone marrow were relatively low at early time points (Supplementary Fig. 9a and 9b) in accordance with their longer half-life (about 2 h). A-EP polymersomes showed a similar uptake level in the liver and the spleen, but a markedly high level of brain and spinal cord uptake compared to pristine EP (Fig. S9c and S9d), suggesting that peptide functionalisation enhanced brain uptake without altering the immune response and clearance. High levels of PMPC-PDPA polymersomes were detected in liver, spleen and bone marrow and the GI tract 15–30 minutes after administration, indicating fast internalisation kinetics (Supplementary Fig. S9e and S9f). This was expected, given the strong affinity of PMPC for SR-B1 and the high expression of these receptors on both immune cells, GI tissues and liver cells. Although lower than for A-EP, PMPC-PDPA polymersomes were also detected in the brain in agreement with SR-B1 brain endothelial expression. The pharmacokinetics of the three polymersome formulations within the brain (Fig. 5e) and spinal cord (Fig. 5e) show more effectively the overall improved performance of the Angiopep-2 peptide. It is interesting to note that the maximum concentration of A-EP in the brain and the spinal cord peaked at 2 hrs and 30 min respectively. Considering that we measured the half-life of A-EP in plasma to be about 2 hrs, these data suggest that there is some mechanism of clearance in both organs which reduces the signal over the time.

The 2D imaging of A-EP in hippocampal sections was subsequently analysed using confocal 3D reconstruction (Fig. 5g, h). As shown in Fig. 5g, 2 hrs after administration functionalised polymersomes were uniformly distributed on the abluminal borders of brain capillaries, indicating ongoing receptor-mediated transcytosis. This polymersome-formed “red border” on capillaries was absent at the 24-hour time-point (Fig. 5h), suggesting complete transcytosis of A-EP. In summary, we have demonstrated the successful delivery of A-EP polymersomes into the CNS, with polymersomes penetrating into the parenchyma.

### Transporting antibody cargo into the CNS using functionalised polymersomes

**Figure 6:**
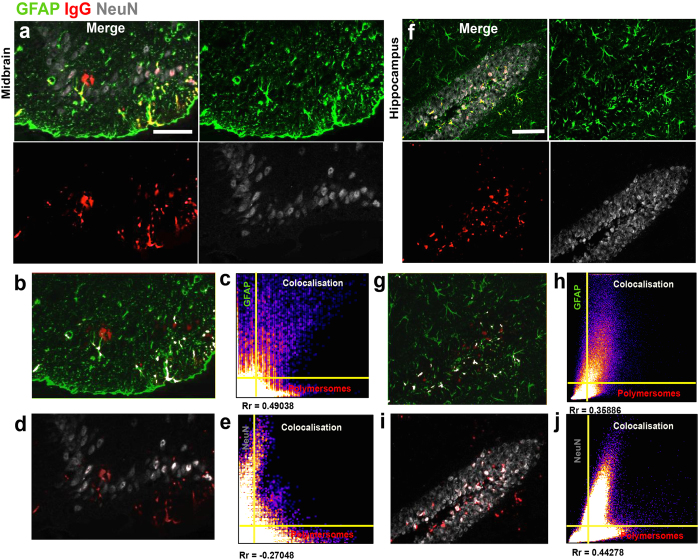
*In vivo* cellular delivery of IgG cargo by A-EP polymersomes. (**a**) and (**f**), Confocal micrographs of midbrain and hippocampus sections from mice after i.v. injection of IgG (red)-loaded A-EP polymersomes, double labelled to show astrocytes (green) and neurons (grey). (**b**) and (**g**), Confocal micrographs of midbrain (**b**) and hippocampus (**g**) sections, with merged staining for IgG and astrocytes. (**d**) and (**i**), Confocal micrograph of midbrain and hippocampus sections, with merged staining for IgG and neurons. (c, e, h, j) Scatter plots of colocalisation profiles of IgG and astrocytes/neurons in sections from midbrain (c, e) and hippocampus (h, j). Scale bars 50 μm.

To establish proof-of-concept for improving delivery of large molecules into the CNS, we next investigated the potential of our A-EP polymersomes to deliver IgG cargo through the BBB. Antibodies (Ab) have been developed for several therapies^42^,^43^,^44^. Recent studies have shown that with the correct affinity, engineered Abs are able to cross the BBB^45^,^46^. However Abs have fast blood clearance, and with few exceptions do not have targeting capability associated with a therapeutic effect, with the consequent need for further chemical modification. An ideal formulation should protect the antibody from potential recognition and degradation during systemic circulation. Therefore, we examined the potential of our pH-sensitive polymersomes to encapsulate an IgG cargo^27^ and deliver it into the cytosol of CNS resident cells^28^. Here we used A-EP as a vector for antibody delivery, and electroporation to encapsulate IgG cargo as reported previously^27^. The antibody-loaded functionalised polymersomes were then administered by tail vein injection in C57BL/6 mice (n = 3), with free IgG acting as a control. Immunostaining for cargo IgG in liver sections 2 h after injection showed less IgG accumulation in functionalised polymersome-treated animals compared to free IgG (Supplementary Fig. S11). The distribution and localisation of cargo IgG was then examined in the brains of mice treated with encapsulated or free IgG, with astrocytes and neurons stained using GFAP and NeuN antibodies respectively. Significant IgG staining was evident throughout the brain following encapsulation, but not following i.v. injection of free IgG (Supplementary Fig. S12). Representative brain sections from the midbrain (or mesencephalon) and hippocampus showing IgG staining are shown in Fig. 6a,f. We then analysed the co-localisation profile (white) between A-EP delivered antibodies and astrocytes (Fig. 6b,g) or neurons (Fig. 6d,i). Some antibody signal was associated with neither astrocytes nor neurons; in the midbrain sections, antibody signals suggested more internalisation by glia (Fig. 6c) than by neurons (Fig. 6e). IgG delivery was clearly observed in the hippocampal slices, showing internalisation by astrocytes (Fig. 6g,h) and neurons (Fig. 6i,j). Taken together, these data demonstrate the *in vivo* transport of antibodies across the BBB using functionalised polymersomes that target LRP-1 receptor-mediated transcytosis. Moreover, encapsulating antibodies in these polymeric nanocarriers allows protection from systemic degradation, and successful delivery into neurons and glial cells of the parenchyma. These results confirm our *in vitro* data and show that the LRP-1 receptor is implicated in two different pathways in endothelial and CNS cells. In the former, it is associated with a fast transcytotic process that does not involve degradation while in the latter it is associated with a more traditional endocytosis process. This enables Angiopep-2-modified pH-sensitive polymersomes to both cross the BBB and target intracellular pathways in CNS cells.

## Conclusions

**Figure 7:**
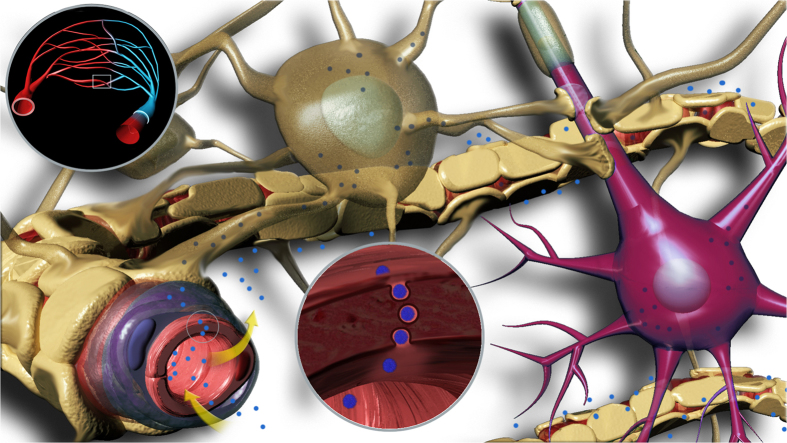
Proposed Mechanism of Polymersome Transcytosis. Schematic representation of ligand-functionalised EP polymersomes crossing the blood-brain barrier (BBB) via receptor-mediated transcytosis and penetrating into brain parenchyma.

The BBB is possibly the most important target for designing new routes to facilitate drug delivery into the CNS. In previous studies, several nanoparticle formulations have been reported to target the BBB and to deliver cargo within the CNS, but so far no mechanism for CNS access and movement within the CNS parenchyma has been suggested. Using a systematic approach to screen nanoparticles *in vitro*, we demonstrate that interaction with relevant endothelial cells is critical but does not in itself guarantee transcytosis. Such cells must be cultured in a 3D model and polarised before transcytosis can be assessed. This is demonstrated using Angiopep-2 and RVG peptides: only the former enabled effective transcellular trafficking, with the latter merely allowing entry into the endothelial cells. More importantly, we show for the first time that LRP-1 mediated transcytosis is a fast process that does not involve endocytic sorting and consequently pH-driven degradation, with the material quickly transported across the endothelial cell layer. Furthermore, the same receptor is also associated with CNS cells, but in this case it drives endocytosis and canonical endocytic sorting with consequent acidification. This means that LRP-1 targeting enables both transport into the CNS and delivery into CNS resident cells. We show this using pH-sensitive polymersomes that (i) are able to encapsulate a high amount of a model payload (IgG) and (ii) release the cargo (IgG) only upon acidification within the early endosomes of CNS cells (see Fig. 7). We prove this both *in vitro* and *in vivo* and provide strong evidence for IgG delivery into CNS cells. This is the first time that IgG has been delivered within CNS cells: this suggests new therapeutic and diagnostic opportunities, exploiting the high affinity of antibodies for relevant intracellular CNS targets. However, many questions remain to be addressed, which were beyond the scope of this article. For example, we must investigate the possibility that the polymersome formulations in this work interact with additional RMT receptors at the BBB. Also, future work should more closely compare the endocytic pathway and transcytosis in bEnd.3 cells, e.g. through a ligand-polymersome combination highly similar to A-EP which instead is targeted for endocytic degradation. The continued use of a 3D co-culture model provides a simple way to gain insight into the process of polymersome transcytosis at the BBB.

## Methods

### Block copolymer synthesis

The 4-(2-bromoisobutyryl ethyl)morpholine initiator (MEBr) was prepared according to a previously published procedure^47^. The protected maleimide initiator was prepared according to a previously published procedure^31^. The Rhodamine 6G-based initiator was prepared according to a previously published procedure^47^. The disulfide-based initiator (BiBOE_2_S_2_) was prepared according to a previously published procedure^48^,^49^. Copper (I) Chloride (CuCl, 99.99%), 2,2’-bipyridine (bpy, 99%), Copper (I) bromide (CuBr, 99.999%), methanol (anhydrous, 99.8%), Poly (ethylene glycol) methyl ether methacrylate (OEG_10_MA), tri-N-butylphosphine (≥93.5%), Biotin-maleimide (≥95%), Biotin (≥99%) and the HABA/Avidin Reagent were purchased from Sigma Aldrich UK (Dorset, UK) and were used as received. The silica gel 60 (0.063–0.200 μm) was purchased from E. Merck (Darmstadt, Germany) and was used as received. HPLC grade chloroform, dichloromethane, ethanol and methanol were obtained from Fisher Scientific (Loughborough, UK) and were used as received. Regenerated cellulose dialysis membranes (1,000 MWCO, 3,500 MWCO and 50,000 MWCO) were from Spectra/Por. Cellulose dialysis membrane (8,000 MWCO) was from BioDesign. 2-(Methacryloyloxy)ethyl phosphorylcholine monomer (MPC, 99.9% purity) was donated by Biocompatibles UK Ltd. (Farnham, UK) and was used as received. 2-(Diisopropylamino)ethyl methacrylate (DPA) was purchased from Scientific Polymer Products (Ontario, US) and passed through an inhibitor removal column (DHR-4, Scientific Polymer Products) prior to use.

### ATRP synthesis of P(OEG_10_MA)_20_-PDPA_100_ from ME-Br, RH-Br and Mal-Br initiators

In a typical procedure, the functional ATRP initiator (0.105 mmol, 1 eq.) was mixed with OEG_10_MA (1 g, 2.11 mmol, 20 eq.). When homogeneous, 1 mL water was added, and the solution was purged with nitrogen for 40 minutes. Then, a mixture of CuCl (10.4 mg, 0.105 mmol) and bpy (32.9 mg, 0.210 mmol) was mixed. After 8 minutes, a sample was removed and a nitrogen-purged mixture of DPA (2.2455 g, 0.0105 mol, 100 eq.) mixed with 3 mL isopropanol was added to the viscous mixture via cannula. After 18 h, the mixture was opened to the atmosphere and diluted with methanol, which gave a dispersion that gradually turned green due to oxidised copper catalyst. Then, 2 volumes of dichloromethane were added, leading to a transparent solution. This was passed through a column of silica using dichloromethane:methanol 2:1 to remove the spent copper catalyst. The resulting solution was dialysed (MWCO 1,000 Da) against ethanol and water. The resulting dispersion was freeze-dried to give a white powder. P(OEG_10_MA)_20_ homopolymer was removed by dialysis against water using dialysis bags with a molecular weight cut-off of 50,000 Da. The resulting copolymer composition was determined by ^1^H NMR in CDCl_3_ and the polydispersity was determined by size exclusion chromatography in THF.

### Deprotection of Mal-P(OEG_10_MA)_20_-PDPA_100_

Deprotection of the protected maleimide-polymer was facilitated by placing the solid purified Mal- P(OEG_10_MA)_20_-PDPA_100_ polymer in a vacuum oven at 100 °C for 15 hours^50^. This led to a slight decolouration and melting of the polymer. The formed maleimide group could not be reliably quantified by ^1^H NMR. Instead, the reactivity of the end-group was demonstrated by its ability to couple to thiol-functional peptides, as assessed by HPLC with fluorescence detection.

### Reaction of Mal-P(OEG_10_MA)_20_-PDPA_100_ with cysteine-terminated peptides Cys-Angiopep and Cys-RVG

The deprotected Mal- P(OEG_10_MA)_20_-PDPA_100_ (105.6 mg, ~3.4 μmol maleimide) was dispersed in 4.5 mL nitrogen-purged PBS at pH 7.3. The pH was lowered by addition of concentrated HCl (10 μL) to give a uniform solution. The pH was then increased to 7.8 with 5 M NaOH and the resulting opaque dispersion was ultrasonicated for 10 minutes. 2.3 mL of this solution was transferred to a 2^nd^ flask. Both solutions were then purged with nitrogen for 10 minutes. (This should give an approximate maleimide amount in each flask of 1.7 μmol). To the original solution was then added Cys-Angiopep (5.5 mg, 2.3 μmol thiol) followed by TCEP (2 mg, 7 μmol). To the 2^nd^ solution was added Cys-RVG (6.0 mg, 1.8 μmol thiol) followed by TCEP (2.3 mg, 8.0 mmol). The pH in each solution was measured to 7. Both solutions were left for 17 h. Then, both solutions were dialysed against water (MWCO 8,000) to remove any excess peptide, followed by freeze-drying. Successful labelling was confirmed using a HPLC with fluorescence and absorption detection: contains fluorescent tyrosine residues, rendering the polymer-peptide conjugates fluorescent at 303 nm when excited at 274 nm. In addition to containing tyrosine, RVG also contains one fluorescent tryptophan residue, which emits at 348 nm when excited at 280 nm. On the other hand, the non-labelled polymer does not exhibit any fluorescence at these wavelengths (but can be detected using the absorption detector).

### Polymersome preparation and physicochemical characterisation

In a typical experiment, 20 mg co polymer (including 10% mol rhodamine-labelled polymer unless otherwise specified) was dissolved in chloroform/methanol (2:1) to a concentration of 3 mg/mL in a glass vial. The solvent was evaporated under vacuum to leave a copolymer film deposited on the walls of the vial. The film was rehydrated using PBS (100 mM, pH 2) to form a 0.5% w/w copolymer suspension, sterilised by filtration (200 nm pore size) before gradually increasing the solution pH to 7.4. Polymersomes were then sonicated for 30–45 min, and purified by gel permeation chromatography (GPC) on a size-exclusion column containing Sepharose 4B. Where polymersomes were to encapsulate cargo or were functionalised with peptides, the solution pH was increased to pH 6.0 ~ 6.3 before cargo was added to the vesicle dispersion, followed by increasing the solution to pH 7.4 and proceeding as above. Dynamic Light Scattering (DLS) was used to assess the polydispersity and average size of polymersome preparations. The machine used was a Malvern Zetasizer. Transmission Electron Microscopy (TEM) was used to assess the size, surface topology and morphology of assembled vesicles. Polymersomes in PBS were deposited onto glow-discharged copper grids at a concentration of 0.5 mg/mL, and stained using 1% phosphotungstic acid (PTA) for 1 minute before drying under vacuum. Images were acquired using a Fei Tecnai G2 Spirit electron microscope, with an acceleration voltage of 80 000 kV.

### *In vitro* 3D cell culture and assessment of barrier properties

For mouse brain endothelial cells (bEnd.3, ATCC® CRL-2299™), the medium used was DMEM supplemented with 10% FCS, penicillin and streptomycin, L-glutamine and Fungizone. Astrocyte (ATCC® CRL-2541™, C8-D1A Astrocyte Type I clone) medium was antibiotics free DMEM supplemented with 10% FCS and L-glutamine. Pericyte (MSC, Gibco®iMouse, C57BL⁄6) medium used was DMEM F12 media with gluta-MAX-I, supplemented with 10% FCS and 5 μg/ml gentamicin. For transwell experiments, both sides of the transwell insert filters (Corning®3460 PE filter, diameter: 1.05 cm, pore size: 0.4μm) were pre-coated with 10 μg/cm^2^ collagen for 2 hours at room temperature. This was followed by seeding bEnd.3 endothelial cells on the upper surface of the transwell at a density of 20,000–40,000 cells/well, and incubated for 12 hours at 37 °C in 95% air 5% CO_2_ in order to allow the cells to fully attach. Next, the astrocytes and/or pericytes (10,000–20,000 cells/well) were seeded on the opposite side of the filter insert, and incubated for 12 hours at 37 °C in 95% air 5% CO_2_. Finally the inserts were moved to a transwell plate, and incubated for 7 days at 37 °C, the medium being changed every two days.

### *In vitro* immunocytochemistry and confocal microscopy of transwell filters

Polymersomes were added at a concentration of 1 mg/ml into the apical (upper) transwell compartment after Trans-Epithelial Electric Resistance (TEER) measurements were taken with an EVOM^2^ Epithelial Voltohmmeter. For the initial uptake experiments, cells were incubated for 3–6 hours at 37 °C in 95% air 5% CO_2_, followed by fixation using 3.7% formaldehyde. Where immunofluorescence was performed, fixation was followed by a 30-minute incubation in 0.3% Triton X-100 and 1% bovine serum albumin (BSA). The transwell insert membrane was excised using a scalpel, and mounted on glass cover slip with VectaShield mounting medium. Cells were imaged on a ZEISS LSM 510 META confocal laser-scanning microscope and Leica SP8 confocal laser-scanning microscope with 40x water immersion lens and 63x oil immersion lens. For rhodamine-labelled polymersomes, an excitation energy 561 nm was used and fluorescence emission was measured at 575–600 nm. Nuclear staining was performed using Hoechst 33342 (500 nM) for 10 min in PBS. Image data was acquired and processed using Zeiss LSM Image Browser, Zeiss LSM Image Expert, Leica and Image J software. The acquisition of co-localisation data by means of Pearson’s correlation coefficient was done via the ImageJ plug-in ‘Colocalization Finder’.

### *Ex vivo* biodistribution

All animal studies were carried out under licence from the UK Home Office, (Scientific Procedures Act 1986) and approved by the University of Sheffield Ethical review committee. The bio distribution study was carried out on six-week-old male C57BL/6 mice (n = 5 per group). Initially, the mice were intravenously injected via the lateral tail vein with 0.1 ml of 10 mg/ml solution of Rhodamine-labelled 1.2% (mol) A-EP, PMPC-PDPA and pristine EP polymersomes. The control group was injected with saline. The mice were humanly terminated by cardiac puncture at pre-set time points (15 minutes, 20 minutes, 2 hours, 4 hours and 24 hours) and then perfused with PBS to remove residual blood from the organs. Subsequently, the organs including liver, spleen, brain and spinal cord were removed and the weight of each organ determined separately. The organs collected were stored at −80 °C for further analysis. The amount of fluorescent signal from Rhodamine 6G (λ_Ex_ = 560 nm, λ_Em_ = 600 nm) in each organ was measured using Xenogen IVIS 100 *in vivo* Imaging System using exposure time of 5 s and a field of view of 4 cm × 4 cm. The intensity of light emission of each organ was quantified as [photons/second]/ [μicrowatt/square centimetre] and normalised by the untreated sample. The radiant efficiency of the control organ was subtracted from the treated organ. The results were then reported as total radiant efficiency ([p/s] / [μW/cm^2^]) (Fig. 5a–c).

### Assessing polymersome penetration into the brain

Three-month-old male C57BL/6 mice were intravenously (i.v.) injected via the tail vein with either Rh-PMPC-PDPA, Rh-EP or Rh-A-EP polymersomes (all n = 6 per group). Control mice were i.v. injected with saline. The volume of solution injected was 8% of the total blood volume (TBV). TBV was calculated as 58.5 mL of blood per kg of body weight. At either 2 hours or 24 hours post-injection (n = 3 for each group), mice were initially injected (i.v.) with 200 μL of fluorescein-labelled lectins (FL-1174 Vector Labs, UK) at 0.5 mg/mL to label the microvasculature *in vivo*. The lectin solution was allowed to circulate for 2 min, following which the mice were terminally anaesthetised and transcardially perfused with phosphate buffered saline (PBS) 0.1 M pH 7.4. Their brains were extracted and the dura mater carefully removed. The tissue was then snap frozen in liquid nitrogen cooled isopentane. Fresh frozen brains from PBS-perfused animals were sectioned at 20 μm in the sagittal plane using a cryostat (Thermo Fisher Scientific, HM-560). Sections were mounted on glass slides, the nuclei stained with 4’,6-diamidino-2-phenylindole dilactate (DAPI; 300 nM in PBS; Sigma, D9564) for 1 min and cover-slipped using an aqueous mountant (Vectashield, Vector Labs, UK). All sagittal brain sections were then manually scanned for rhodamine florescence using a stereomicroscope fitted with a 20:1 zoom range to allow efficient macro and micro viewing (M205FA, Leica). Higher resolution microscopy was performed on areas of interest using a Zeiss LSM 510 confocal system using a 40x magnification IR Zeiss dipping objective (0.8 NA). DAPI (λ_Exc_ = 405, λ_Em_ = 420–450), Lectub (λ_Exc_ = 488, λ_Em_ = 500–550), polymersomes (λ_Exc_ = 548, λ_Em_ = 560–600). Image data was acquired and processed using Zeiss LSM Image Browser, Zeiss LSM Image Expert and ImageJ software.

### Assessing IgG cargo delivery into the brain

Three-month-old male C57BL/6 mice were i.v. injected via the tail vein with either EP (n = 3) or A-EP (n = 3) polymersomes carrying cargo IgG (Abcam). Free cargo IgG was administered i.v. into C57BL/6 mice, which acted as a control (n = 3). Two hours after i.v. injection, mice were anaesthetised and transcardially perfused with PBS, followed by 4% (w/v) paraformaldehyde (PFA) in PBS. The perfused brains were extracted, dura mater removed, then post-fixed for 7 hours in 4% PFA at 4 °C. The fixed brains were immersed in 20% (w/v) sucrose in PBS overnight at 4 °C for cryoprotection. Fixed brains were cut using a cryostat at 20 μm in the coronal plane and mounted on glass slides. For astrocyte and neuronal immunostaining, sections were initially washed three times for 5 min in PBS at room temperature and pre-incubated for 1 hour in antibody-blocking buffer consisting of 2% goat serum and 1% bovine serum albumin (BSA) in 0.1% PBS-Triton X-100 (PBST). After washing with PBS, processed sections were incubated with primary antibodies diluted in PBS for 1 hr at room temperature: polyclonal rabbit anti-GFAP (Dako; 1:500), mouse anti-NeuN (Millipore; 1:500). Sections were washed four times in PBS for 5 min each. Sections were then incubated for 2 h at room temperature using the following secondary antibodies: goat anti-rabbit Alexa 488 (1:200), goat anti-mouse Alexa 647 (1:200) both produced by Molecular Probes (Life Technologies). To detect the presence of IgG cargo in brain, we used a secondary antibody conjugated to Dylight 549 (1:500; Abcam). After intensive washing in PBS, the sections were cover-slipped (0.17 mm) following application of Vectashield mounting medium. Slides were then imaged using laser-scanning confocal microscopy (Zeiss LSM 510) for emission of GFAP (λ_Exc_ = 488, λ_Em_ = 500–550), Dylight 549 (λ_Exc_ = 548, λ_Em_ = 560–600), NeuN (λ_Ecv_ = 647, λ_Em_ = 650–700). Image data was acquired and processed using Zeiss LSM Image Browser, Zeiss LSM Image Expert and ImageJ software.

### Animals

All procedures involving animals were approved by and conformed to the guidelines of the Institutional Animal Care Committee of The University of Sheffield. We have taken great efforts to reduce the number of animal used in these studies and also taken effort to reduce animal suffering from pain and discomfort.

## Additional Information

**How to cite this article**: Tian, X. *et al.* LRP-1-mediated intracellular antibody delivery to the Central Nervous System. *Sci. Rep.* **5**, 11990; doi: 10.1038/srep11990 (2015).

## Supplementary Material

Supplementary Information
