# Supplementary material for: LRP-1-mediated intracellular antibody delivery to the Central Nervous System
Source: Sci Rep. 2015 Jul 20;5:11990. doi: 10.1038/srep11990 (PMC4507173; doi:10.1038/srep11990)
Supplement: Supplementary Information [file srep11990-s1.pdf]

Supplementary Information

LRP1-mediated intracellular antibody delivery to the Central Nervous System

Xiaohe Tian[1,2,§] Sophie Nyberg[1,2,§] Paul Sharp[3,5,6], Jeppe Madsen[3,4], Nooshin Daneshpour[3,5,6], Steven P. Armes[4], Jason Berwick[7], Mimoun Azzouz[5,6], Pamela Shaw[5,6], N. Joan Abbott[8] & Giuseppe Battaglia[1,2]

[1]Department of Chemistry, [2]The MRC/UCL Centre for Medical Molecular Virology, University College London, London, UK. [3]Department of Biomedical Science, [4]Department of Chemistry, [5]Sheffield Institute for Translational Neuroscience (SITraN) [6]Department of Neuroscience, [7]Department of Psychology, University of Sheffield, Sheffield, UK. [8]Institute of Pharmaceutical Science, King's College London

Correspondence should be addressed to G.B. (g.battaglia@ucl.ac.uk) Department of Chemistry, University College London, London, 20 Gordon Street, WC1H 0AJ London, UK. Tel.

[§]**These authors contributed equally.**

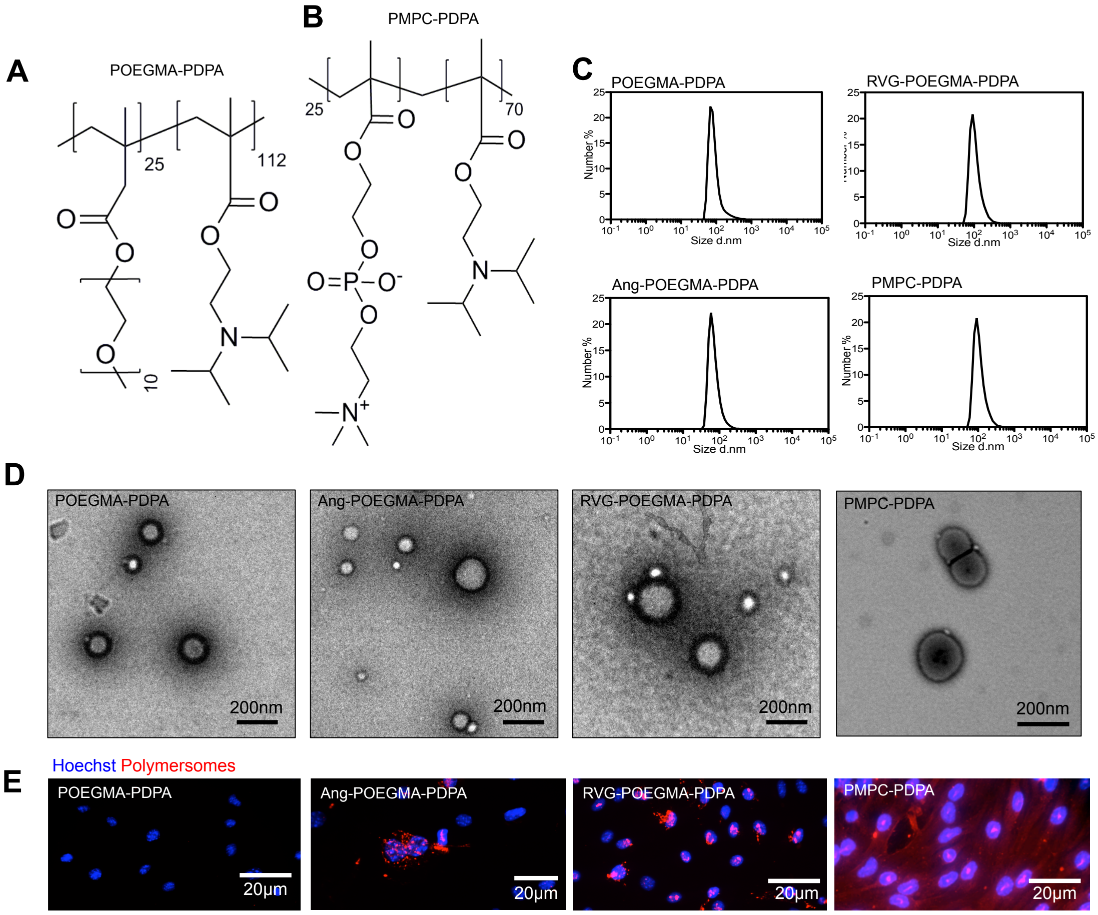

Fig. S1. A and B, Chemical structure of $P(OE)_{10}GMA_{25}$-$PDPA_{112}$ and $PMPC_{25}$-$PDPA_{70}$. C, Dynamic light scattering determination of polymersome size distribution. D, Transmission electron micrographs showing the vesicular morphology of the polymersomes. E, Uptake of polymersomes by bEnd.3 cells in 2D culture (2 hours).

Z-Potential

Polymersomes	PMCP	PEO	RVG-PMPC	Ang-PMPC	PEOGMA
Zeta Potential / mV (T=25.1°C)	-0.990±0.13	-0.69±0.41	-2.011±0.37	-1.978±0.25	-0.795±0.42

Continued

Ang-PEOGMA	RVG-PEOGMA
-1.125±0.73	-2.013±0.69

Fig. S2. Electrical properties of polymersomes indicated by Zeta-potential measurements.

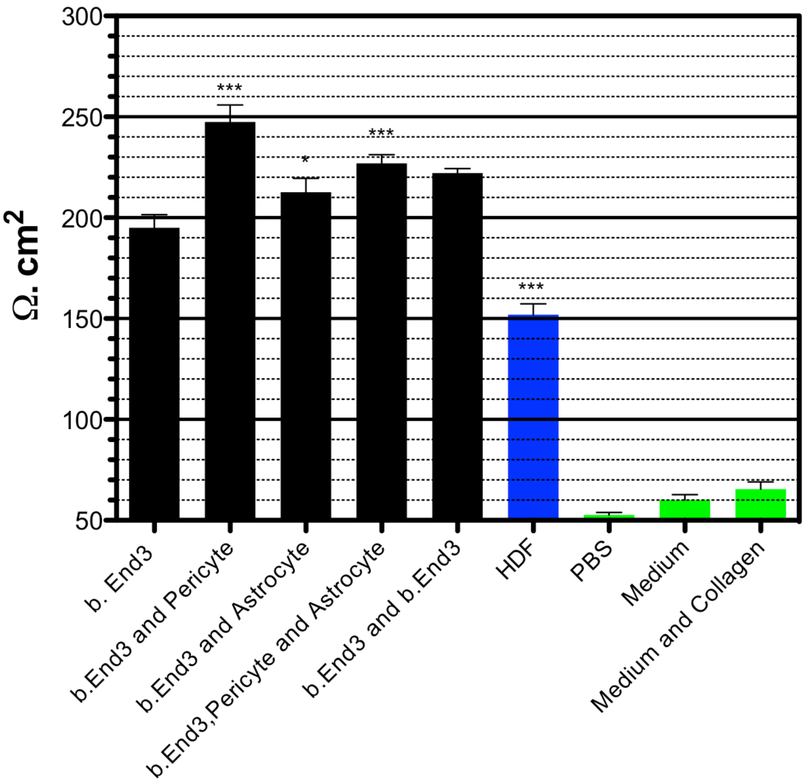

Fig. S3. Trans-endothelial electrical resistance measurement (TEER) of the *in vitro* BBB model in different co-culture conditions, 7 days incubation. Error bar: SEM, =3. * p<0.05, ***p<0.001

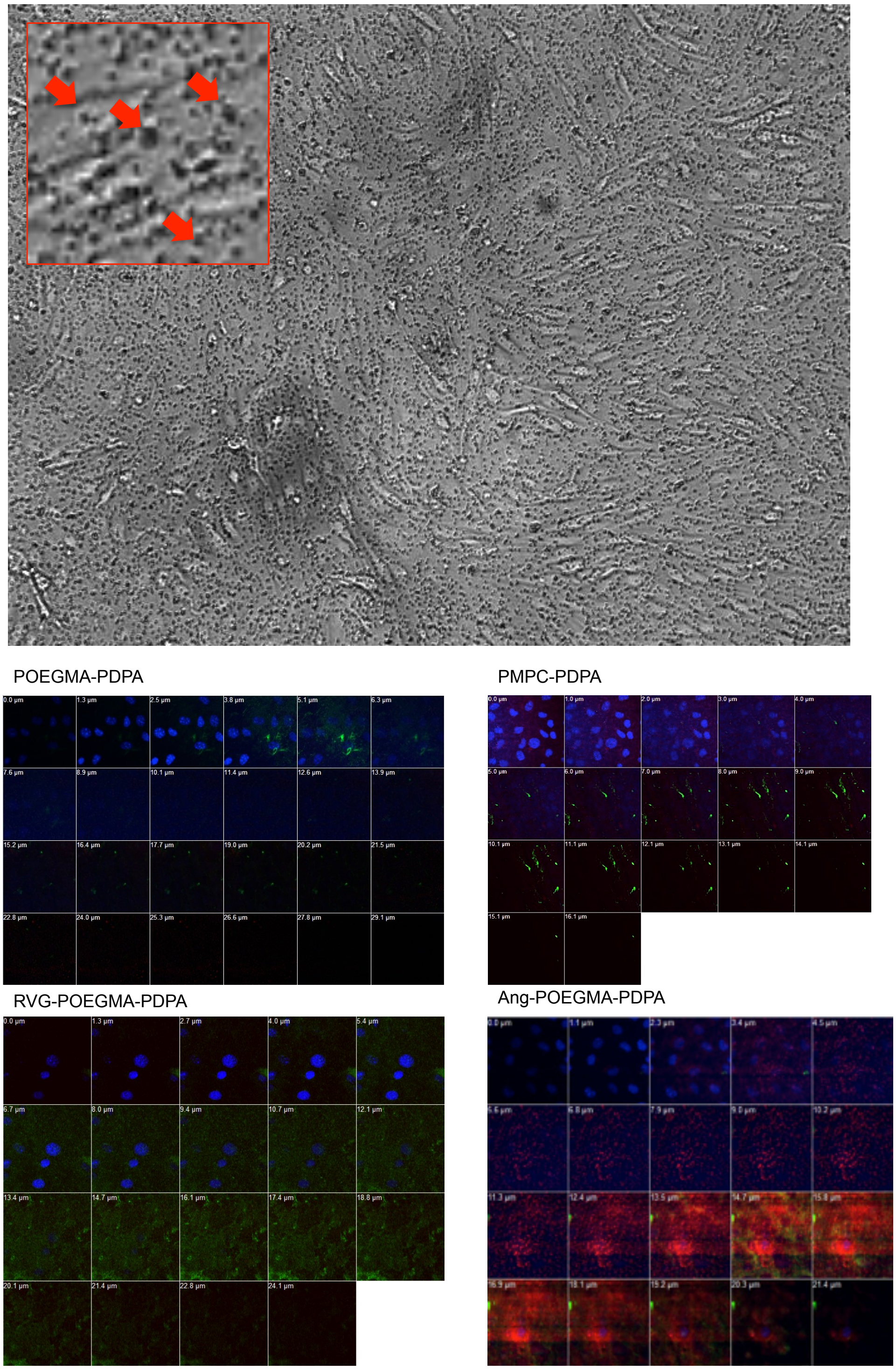

Fig. S4. Transmission light micrograph showing confluent bEnd.3 cell monolayer on transwell insert filter, and gallery micrographs from Figure 1c. Red arrows indicate pores in the transwell filter membrane.

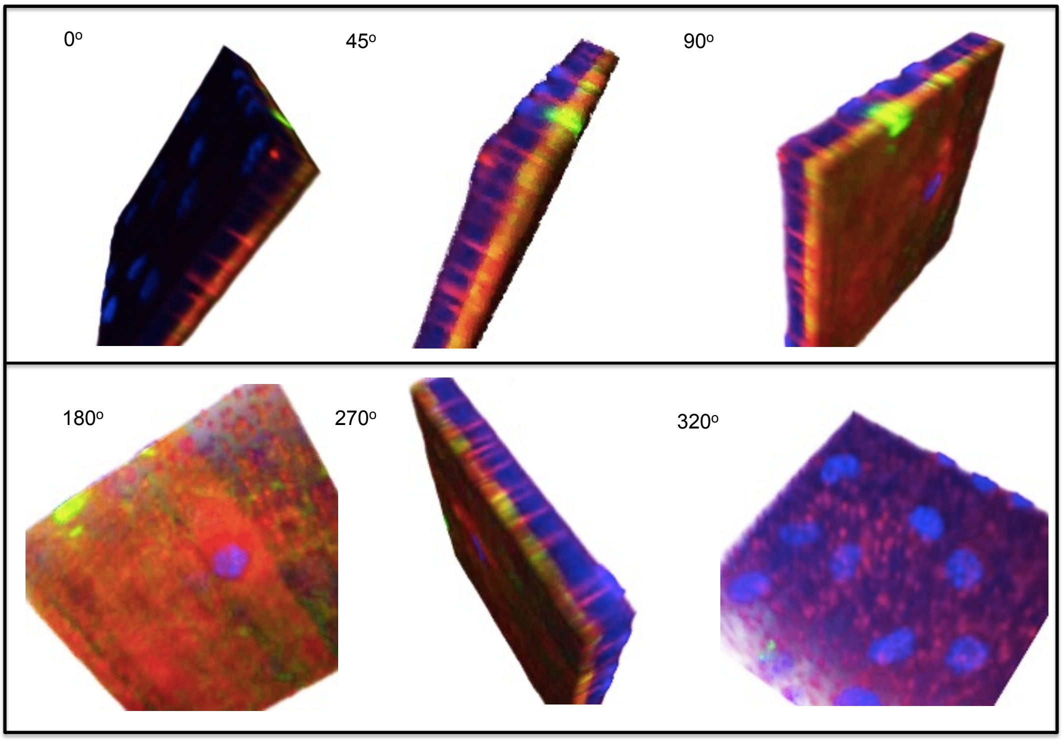

Fig. S5. 3D reconstructed, 360 degree rotation of the 3D *in vitro* BBB model (bEnd.3 cells) grown on transwell filter membrane treated with A-EP polymersomes for 2 hours.

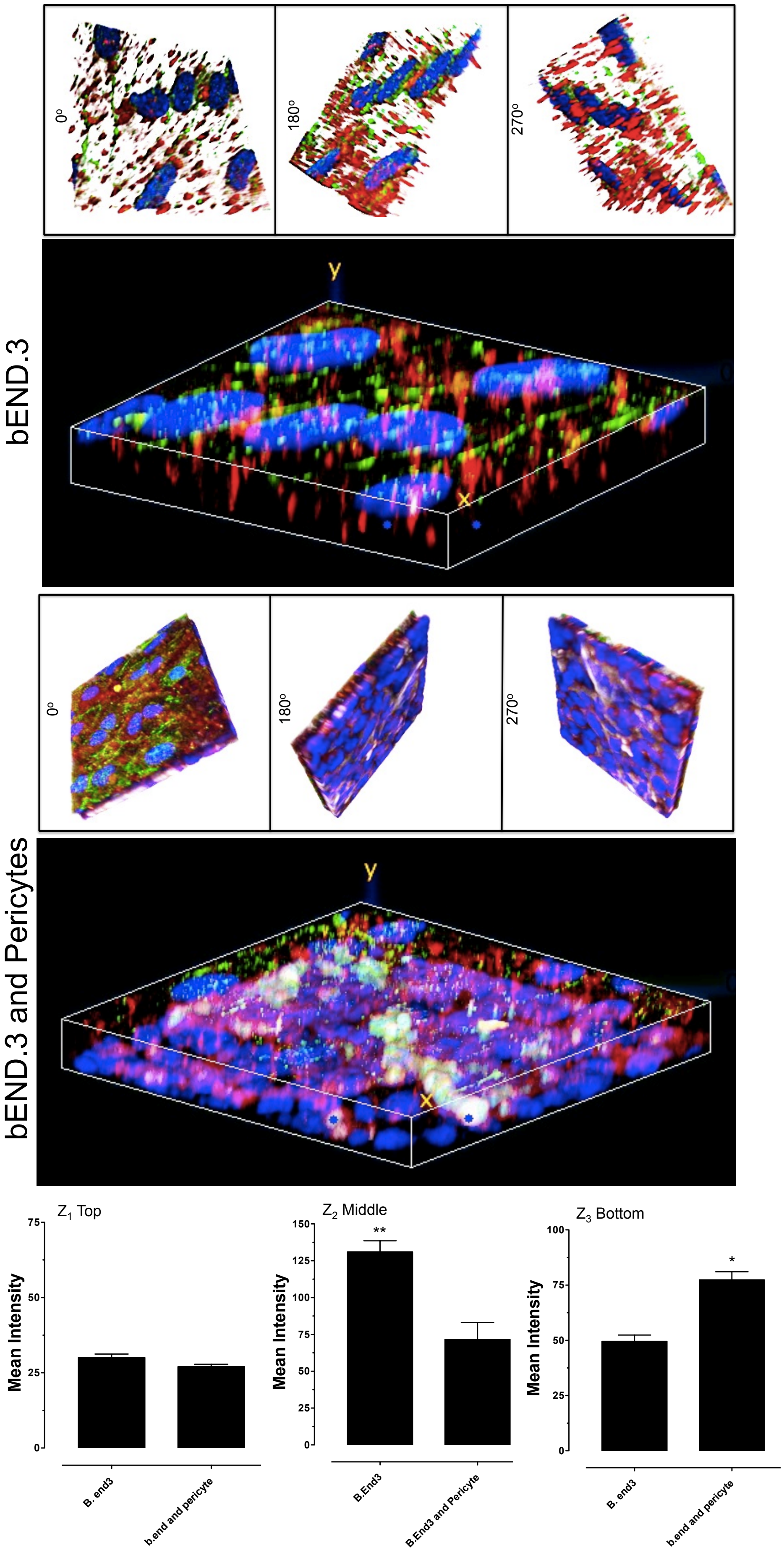

bEND.3
0°
180°
270°
y
x
bEND.3 and Pericytes
Z1 Top
Z2 Middle
Z3 Bottom
Mean Intensity
B. end3
b.end and pericyte
B.End3
B.End3 and Pericyte
**
*

Fig. S6. 3D reconstructed, 360 degree rotation of the 3D *in vitro* BBB models (bEnd.3 and co-cultured bEnd.3/Pericytes) treated with A-EP polymersomes for 2 hours. Lower panel: Fluorescence intensity analysis from top, middle and bottom sections of transwell filter.

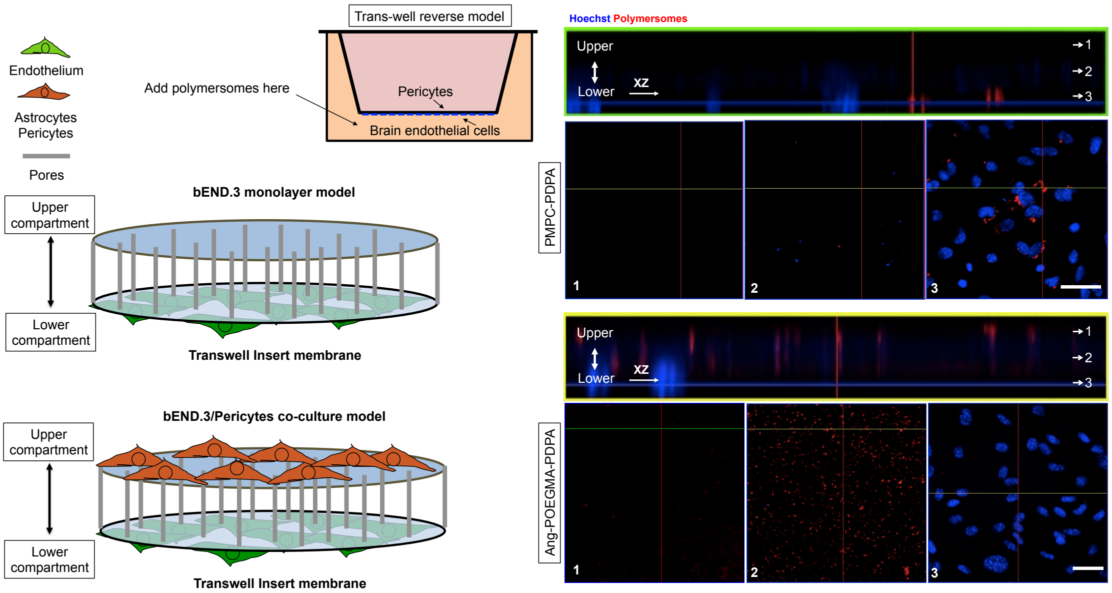

Fig. S7. Schematic representation of the 'reverse' BBB transwell model and 3D/ cross-section micrographs from MD and A-EP polymersome treated (2 hours) experiments . Scale bars=20µm

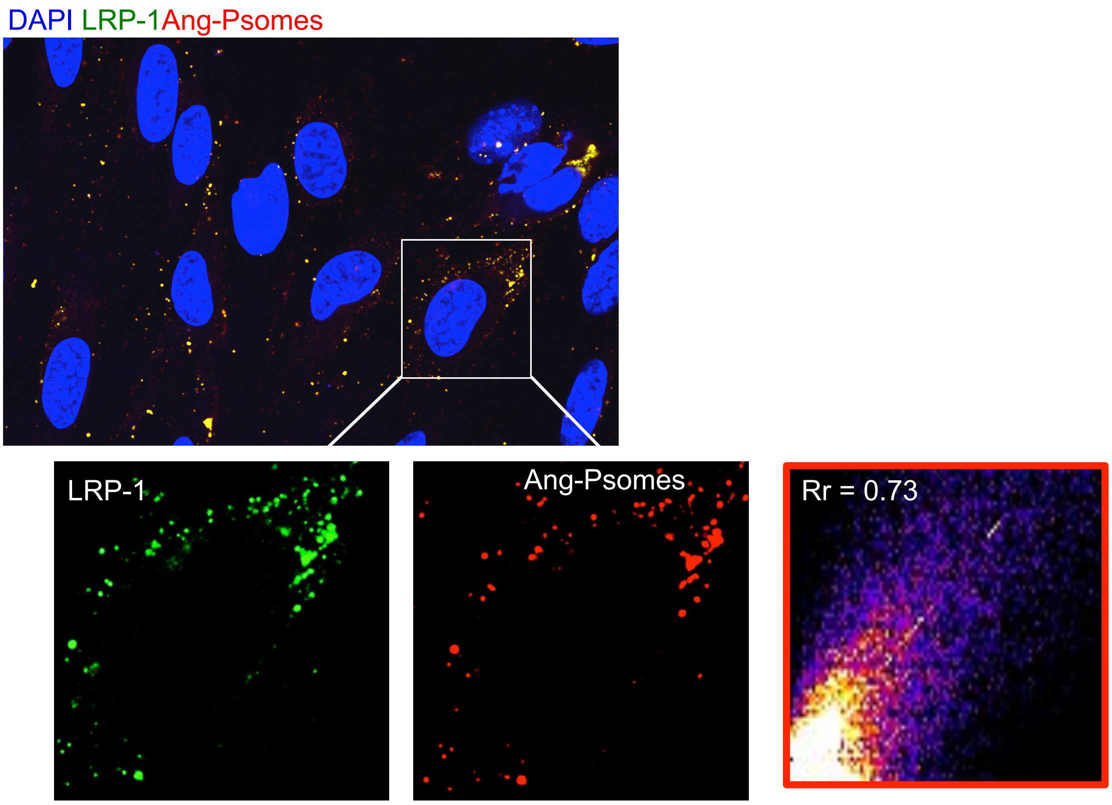

Fig. S8. Co-staining LRP-1 antibody with A-EP polymersomes in bEnd.3 cell monolayer on transwell filter after 2 hours treatment. Pearson's colocalisation coefficient: 0.73.

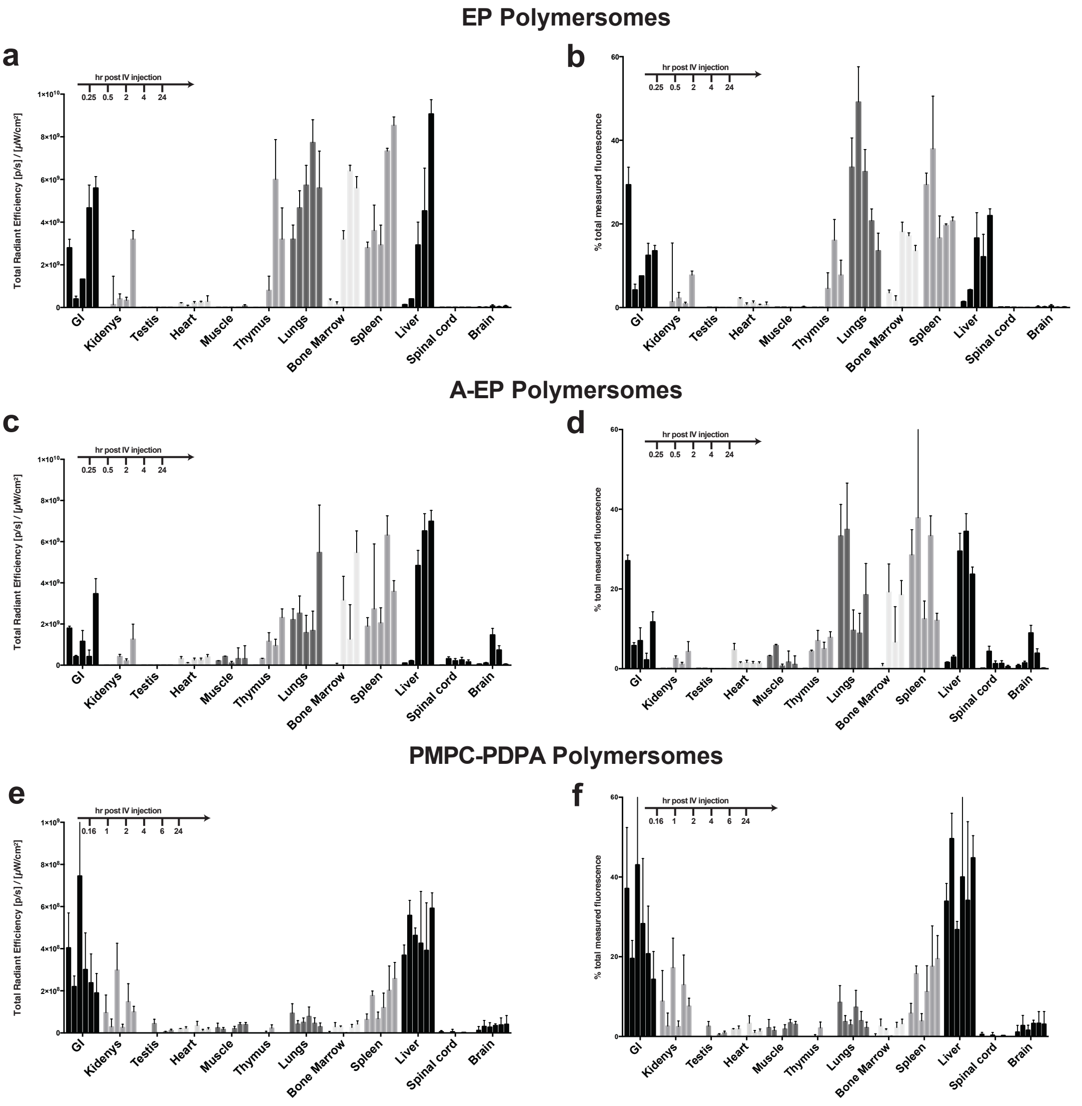

Fig. S9. *Ex vivo* quantitative fluorescence imaging using IVIS spectrum. Histograms showing both absolute Total Radiant Efficiency (TRE) [p/s] / [μW/cm^2] and normalized TRE for total signal across all the organs analysed for EP (**a, b**), A-EP (**c,d**) and PMPC-PDPA polymersomes (**e,f**) at different times points after I.V. Injection

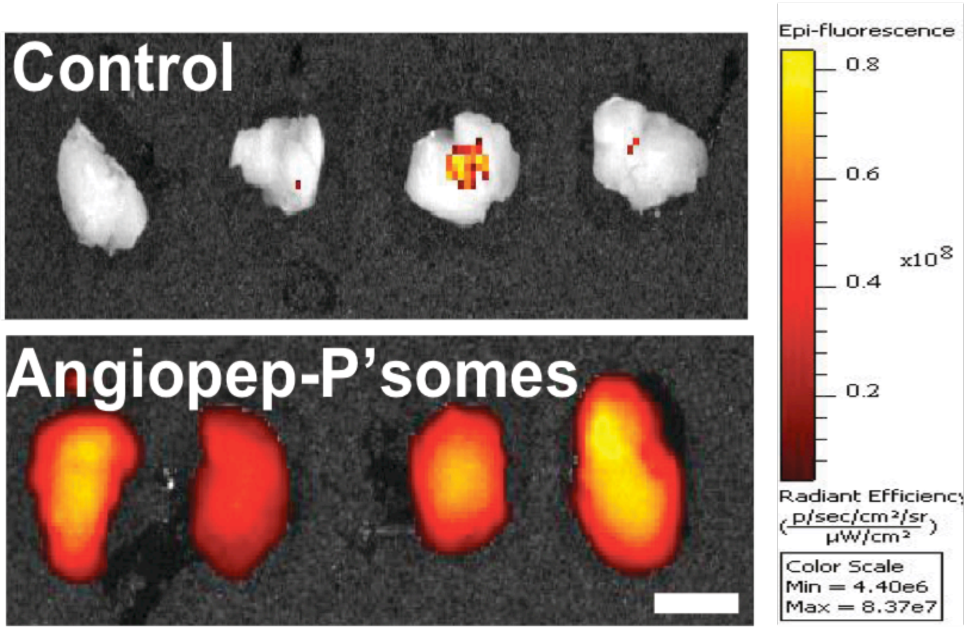

Figure S10. *Ex vivo* fluorescence imaging of brains using IVIS. Raw images of mouse brains in n=4 independent experiments, showing radiant efficiency in control brains (top) and A-EP polymersomes (bottom).

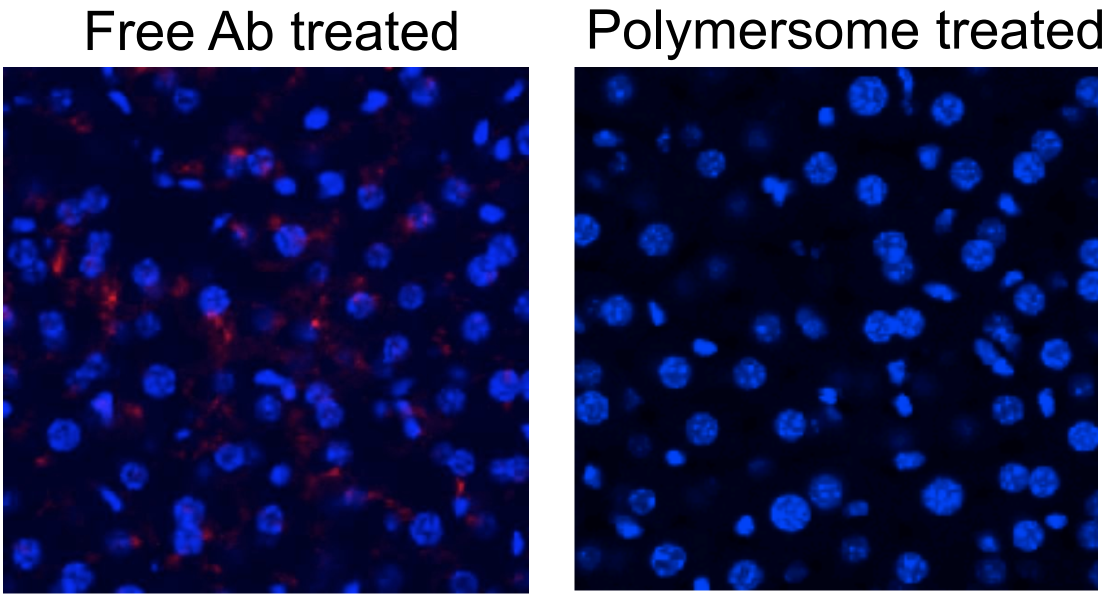

Fig. S11. Confocal micrographs of liver sections from mice 2 hours after i.v. injection with free IgG or IgG-loaded polymersomes.

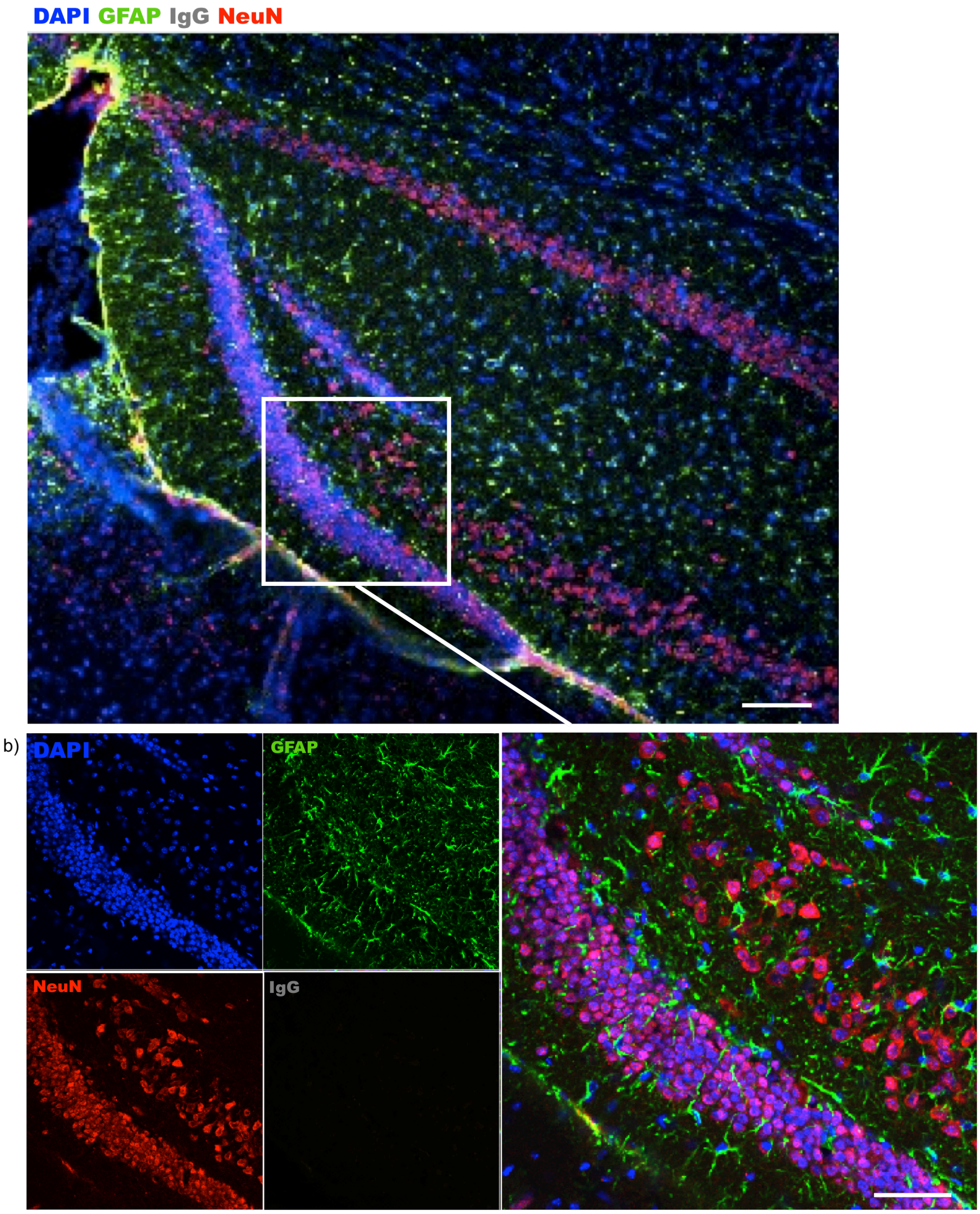

Fig. S12. Confocal micrographs showing representative brain hippocampus section from mouse 2 hours after injection (i.v.) with free IgG.
